# Supplementary material for: Complex‐centric proteome profiling by SEC‐SWATH‐MS
Source: Mol Syst Biol. 2019 Jan 14;15(1):e8438. doi: 10.15252/msb.20188438 (PMC6346213; doi:10.15252/msb.20188438)
Supplement: Supplementary file 6 — Dataset EV5 [file MSB-15-e8438-s006.zip › feature_plots_corum/3137-1.pdf]

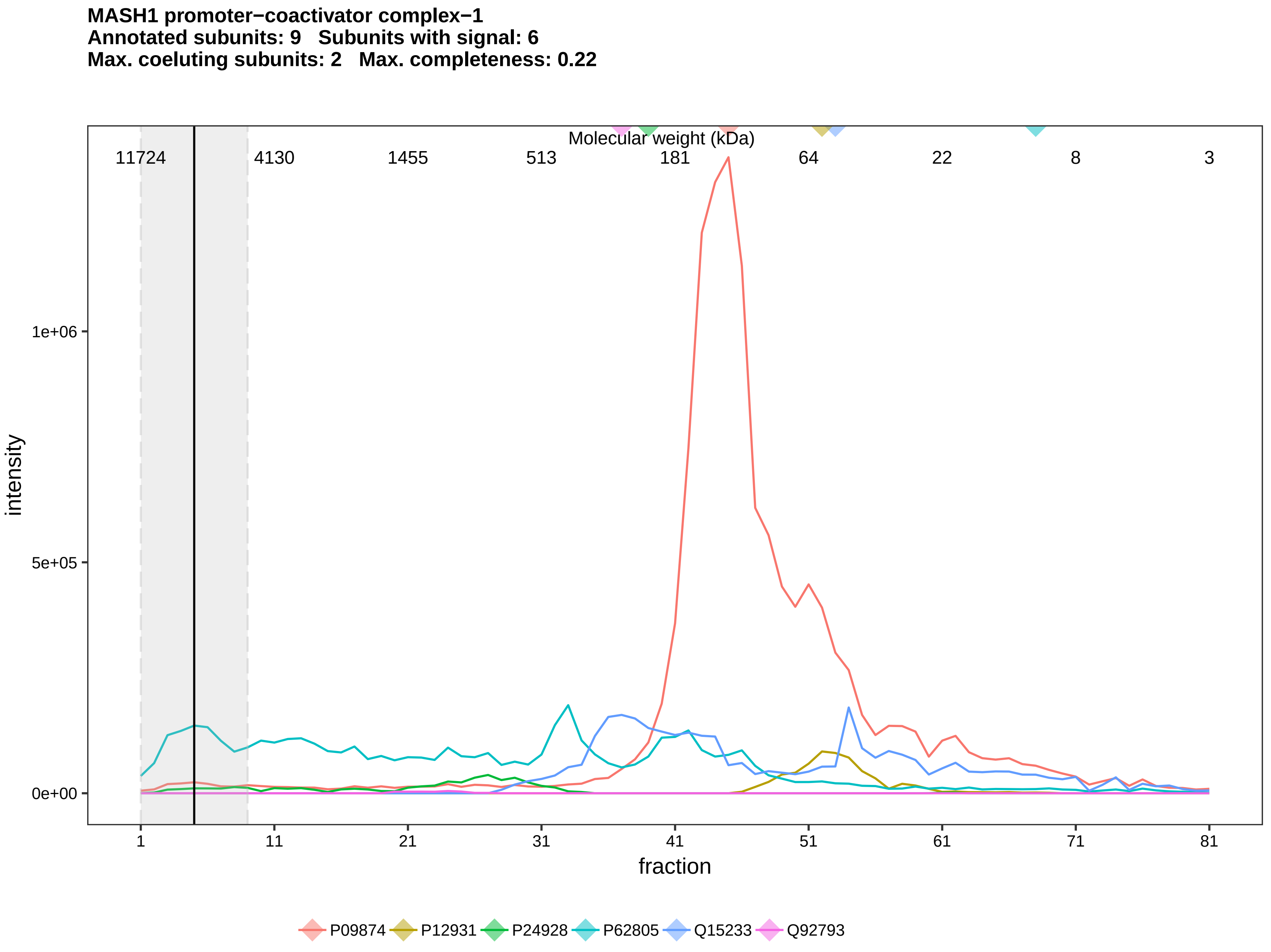
MASH1 promoter−coactivator complex−1
Annotated subunits: 9   Subunits with signal: 6
Max. coeluting subunits: 2   Max. completeness: 0.22
Molecular weight (kDa)
11724
4130
1455
513
181
64
22
8
3
1e+06
5e+05
0e+00
intensity
1
11
21
31
41
51
61
71
81
fraction
P09874
P12931
P24928
P62805
Q15233
Q92793
